# Acidic Compartment Size, Positioning, and Function during Myogenesis and Their Modulation by the Wnt/Beta-Catenin Pathway

**DOI:** 10.1155/2020/6404230

**Published:** 2020-06-20

**Authors:** Kayo M. Bagri, Ivone A. Rosa, Stephany Corrêa, Aline Yamashita, José Brito, Flavia Bloise, Manoel Luis Costa, Claudia Mermelstein

**Affiliations:** ^1^Instituto de Ciências Biomédicas, UFRJ, RJ, Brazil; ^2^Instituto Nacional de Câncer, RJ, Brazil; ^3^Instituto de Bioquímica Médica Leopoldo de Meiss, UFRJ, RJ, Brazil; ^4^Instituto de Biofísica Carlos Chagas Filho, UFRJ, RJ, Brazil

## Abstract

Lysosomes and acidic compartments are involved in breaking down of macromolecules, membrane recycling, and regulation of signaling pathways. Here, we analyzed the role of acidic compartments during muscle differentiation and the involvement of the Wnt/beta-catenin pathway in lysosomal function during myogenesis. Acridine orange was used to localize and quantify acidic cellular compartments in primary cultures of embryonic muscle cells from *Gallus gallus*. Our results show an increase in acidic compartment size and area, as well as changes in their positioning during the initial steps of myogenesis. The inhibition of lysosomal function by either the chloroquine Lys05 or the downregulation of LAMP-2 with siRNA impaired chick myogenesis, by inhibiting myoblast fusion. Two activators of the Wnt/beta-catenin pathway, BIO and Wnt3a, were able to rescue the inhibitory effects of Lys05 in myogenesis. These results suggest a new role for the Wnt/beta-catenin pathway in the regulation of acidic compartment size, positioning, and function in muscle cells.

## 1. Introduction

The formation of skeletal muscle fibers involves the biogenesis of new organelles and the recycling of damaged organelles. Previous work has shown that degradative processes are essential in the differentiation of myoblasts and in the formation of mature myotubes [1, 2]. Although several degradative systems, such as the ubiquitin-proteasome system, calpains, and caspases, are involved in myoblast differentiation, skeletal muscle formation requires significant morphological changes and organelle and membrane remodeling, which may not be fully supported by these processes and may require the participation of the lysosomal system. Acidic compartments, such as lysosomes, are involved in the recycling of macromolecules and cell membrane repair, among many functions. They are heterogeneous in size, number, shape, degradative activity, luminal pH, and subcellular distribution, and these parameters can be finely modulated in complex cellular events, such as cell differentiation [3].

Recently, lysosomes and other acidic compartments have emerged as dynamic and sophisticated signaling centers that can govern cell growth, division, and differentiation [4]. The lysosome surface serves as a platform to assemble major signaling hubs like mTORC1, AMPK, and GSK3*β* [3]. It has been shown that GSK3*β* localization to lysosomes promotes cell survival and growth, while its localization to the nucleus promotes cell death in human epithelial cell lines [5]. GSK3*β* is a kinase with multiple putative substrates, including beta-catenin, a key effector protein in the Wnt/beta-catenin signaling pathway. Wnt/beta-catenin is one of the major pathways controlling muscle proliferation and differentiation [6–8].

A limited number of studies focused on the role of acidic compartments during skeletal myogenesis. Sakane and Akasaki [9] have shown that the downregulation of lysosome-associated membrane proteins LAMP-1 or LAMP-2 impairs the differentiation of C2C12 mouse myoblasts and reduces the diameter of myotubes. LAMP-2 knockdown more severely impaired C2C12 myotube formation compared with LAMP-1 knockdown. Ebisui and colleagues [2] demonstrated that the expression and activity of lysosomal cathepsins increase during differentiation of myoblasts into myotubes. It was also reported that LAMP-2 knockout mice exhibit autophagic vacuole accumulation in skeletal muscles [10].

The lack of studies on the role of acidic compartments in chick myogenesis, and particularly in their involvement in the regulation of signaling pathways during skeletal myogenesis, prompted us to characterize (i) the size of acidic compartments during muscle differentiation, (ii) the effects of lysosomal inhibition by the chloroquine Lys05 during myogenesis, (iii) the effects of LAMP-2 knockdown during myogenesis, and (iv) the relationship between acidic compartments and the Wnt/beta-catenin pathway during chick *in vitro* myogenesis.

## 2. Materials and Methods

### 2.1. Myogenic Cell Culture

This study using chick embryos was approved (069/19) by the Ethics Committee for Animal Care and Use in Scientific Research from the Federal University of Rio de Janeiro (Brazil). Primary cultures of myogenic cells were prepared from breast muscles of embryonic day 11 (E11) *Gallus gallus* embryos, as previously described [11]. For lysosomal inhibition, 24-hour cells were treated with the chloroquine Lys05 (Sigma, dissolved in 0.1% DMSO) at a final concentration of 0.05, 0.5, 1, and 5 *μ*M or with 0.1% DMSO, both for 24 hours. For the activation of the Wnt/beta-catenin pathway, 24-hour cells were treated with 5 *μ*M 6-bromoindirubin-30-oxime (BIO, Sigma) or conditioned media (50% *v*/*v*) enriched in Wnt3a (obtained from L-Wnt3a cells, ATCC, USA).

### 2.2. LAMP-2 Knockdown

siRNA sequences specific for LAMP-2 of *Gallus gallus* (mRNA sequence obtained from NCBI reference sequence NM_001030295) or enhanced green fluorescent protein (eGFP) were from Integrated DNA Technologies (USA), as previously described [12]. The *Gallus gallus* sequences of siRNA that were selected to target LAMP-2 or eGFP are the following:

(1) eGFP from *Gallus gallus*:
  - 5′rArArGrCrUrGrArCrCrCrUrGrArArGrUrUrCrArUrCrUrGCA 3′ (strand)
  - 5′rUrGrCrArGrArUrGrArArCrUrUrCrArGrGrGrUrCrArGrCrUrUrGrC 3′ (passenger)
(2) LAMP-2 from *Gallus gallus* (seq 1467-1492):
  - Sense 5′ rGrArArUrUrGrGrArArArUrUrUrArCrUrUrGrArArGrCrUAC 3′
  - Antisense 5′ rGrUrArGrCrUrUrCrArArGrUrArArArUrUrUrCrCrArArUrUrCrArU 3′

### 2.3. Immunofluorescence

Cells were fixed with 4% paraformaldehyde, permeabilized with 0.5% Triton-X 100, and incubated with anti-desmin antibodies (Sigma) followed by Alexa Fluor-conjugated secondary antibodies and DAPI (0.1 *μ*g/ml) and examined with an Axiovert 100 microscope (Carl Zeiss).

### 2.4. Acidic Compartment Labeling with Acridine Orange

24-hour cultures were treated with Lys05 for the next 24 hours. 48-hour cells were stained with 2 *μ*g/ml acridine orange solution for 10 min at 37°C and 5% CO_2_. Cells were examined with an Axiovert 100 microscope. The diameter and area of acidic compartments, as well as the distance between acidic compartments and the nuclei of each cell, were quantified using Fiji software. The area occupied by acidic compartments per cell was measured in relation to the total cell area (area of acidic compartments (%) ÷ total cell area (%)).

### 2.5. Quantification of Chick Cell Cultures

Chick myogenic cultures were double-labeled for desmin (a muscle-specific marker) and DAPI, and merged images were used for counting the presence of nuclei within mononucleated or multinucleated cells using Fiji software or CellProfiler Analyst [13] (http://cellprofiler.org). The DAPI labeling enables the identification of myoblasts and fibroblasts by their nuclear morphologies and fluorescence intensities: muscle fibroblasts have large, flattened, and pale nuclei whereas myoblasts have small, round, and bright nuclei.

### 2.6. SDS-PAGE and Immunoblotting

Total extract from myogenic cells was prepared, and equal amounts of protein were loaded onto 12% SDS-polyacrylamide gels. Following electrophoresis, proteins were transferred to PVDF membranes and incubated with anti-LAMP2 (Thermo Fisher, USA) or anti alpha-tubulin (Sigma) antibodies. Protein bands were visualized using an Odyssey Infrared Imager (LI-COR Biosciences, USA).

### 2.7. qPCR

For the analysis of mRNA expression of the genes LAMP-1, LAMP-2, FLOT-1 (flotillin-1), FLOT-2 (flotillin-2), DES (desmin), and MYOG (myogenin) from *Gallus gallus*, cells were treated with DMSO or 1 *μ*M Lys05. GAPDH mRNA expression was similar between the groups; thus, it was chosen as the reference gene. The PCR program was as follows: denaturation 12 min 95°C, 40 cycles of 15 sec 95°C, 30 sec 60°C, and 30 sec 72°C, following the melting program. Quality of qPCR and genomic DNA contamination were checked using intron-spanning primers, reverse transcriptase-negative samples from cDNA synthesis, and melting curve analysis obtained from each reaction. Sequences of primers are described in Supp. Fig. 1.

### 2.8. Statistical Analysis

One-way ANOVA followed by Dunnett's multiple comparisons test was used for myogenesis parameters, and Tukey's multiple comparisons test or Student *t*-test for qPCR. A *P* value of less than 0.05 was considered statistically significant. Results are represented as the mean ± standard deviation of 7 independent experiments. ^∗^*P* ≤ 0.05, ^∗∗^*P* ≤ 0.01, and ^∗∗∗^*P* ≤ 0.001.

## 3. Results

### 3.1. Acidic Compartment Diameter and Area Changes during Chick Myogenesis

**Figure 1:**
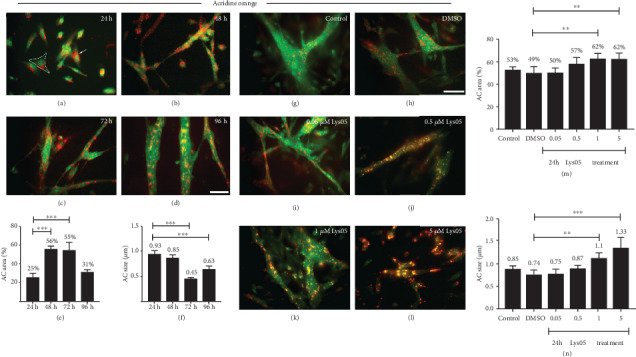
Characterization of acidic compartments in muscle cells. Chick myogenic cells were labeled with acridine orange, analyzed under a fluorescent microscope (a–d, g–l). Control (untreated) cells were grown for 24, 48, 72, or 96 hours (a–d). Other cells were grown for 24 hours, treated with DMSO, or Lys05-treated for the next 24 hours (g–l). Acidic compartments (AC) were found specially concentrated in the perinuclear area of mononucleated cells (arrow in (a)). White outline shows how the cell surface was measured (a). The area and diameter of acidic compartments were quantified (e, f, m, and n). Scale bar = 50 *μ*m.

To understand the role of acidic compartments during chick myogenesis, we first analyzed the total area occupied by acidic compartments and their diameter in chick muscle cells grown *in vitro* for 24, 48, 72, and 96 hours (Figures 1(a)–1(d)). These different time points reflect different developmental stages of chick myogenesis. We used acridine orange, a cell-permeable fluorescent cationic dye, to visualize acidic compartments. Acridine orange emits green fluorescence when bound to DNA or RNA and red fluorescence when bound to acidic compartments. Our results show that all cell phenotypes found in chick muscle cell cultures (fibroblasts, myoblasts, and myotubes) were labeled in red (acidic compartments) and green (cytoplasm and nuclei) with acridine orange (Figures 1(a)–1(d)). The green cytoplasmic stain was used for the comparison between the area occupied by acidic compartments (in red) and the entire area of the cell (in green). Acidic compartments were especially concentrated in the perinuclear area in mononucleated cells (myoblasts and fibroblasts) from 24 h cultures (Figure 1(a)). We found an increase in the area occupied by acidic compartments in 48 and 72 h cultures, in comparison with 24 h cultures (Figure 1(e)). The diameter of acidic compartments varied between 0.45 and 0.93 *μ*m, with the largest acidic compartments found in 24 and 48 h cultures (Figure 1(f)). A detailed observation of the fluorescence images from Lys05-treated cells compared to untreated and DMSO-treated cells showed that the red labeling of acridine orange was reduced in cellular compartments after Lys05 treatment, while a yellow labeling of acridine orange increased (Figures 1(g)–1(l)). The yellow labeling of acridine orange suggests a decrease in red (more acidic) labeling and an increase in green (less acidic) labeling of cellular compartments. Therefore, these experiments show that Lys05 is inducing the deacidification of acidic compartments in chick muscle cells while inducing an increase in the size and area of the acridine orange-labeled cellular compartments.

### 3.2. Inhibition of Lysosomal Enzymes Alters Acidic Compartments Diameter and Area in Muscle Cells

The variation in the diameter and total area of acidic compartments observed in the 24-96-hour muscle culture period suggested a role for acidic compartments during embryonic chick myogenesis. To investigate this hypothesis, we decided to inhibit acidic compartment function and analyze its effects on chick muscle cultures. We used different concentrations of Lys05, a dimeric chloroquine that deacidifies acidic compartments and causes impairment of acidic compartment enzymes. Acridine orange was used to visualize and quantify acidic compartments in Lys05-treated cells. No significant effects in the acidic compartment area and diameter were detected in cells with Lys05 at the concentrations of 0.05 and 0.5 *μ*M (Figures 1(m) and 1(n)), whereas 1 and 5 *μ*M of Lys05 caused an increase in the area occupied by acidic compartments (27% compared to DMSO) and in the diameter of acidic compartments (29% and 56%, respectively). The average diameter of acidic compartments in untreated cultures was 0.85 *μ*m, in DMSO-treated cultures was 0.74 *μ*m, and in Lys05-treated cultures ranged between 0.75 and 1.33 *μ*m (Figure 1(n)). These results show that inhibition of lysosomal enzymes leads to an increase in the area and diameter of acidic compartments in chick muscle cells.

### 3.3. Inhibition of Acidic Compartment Enzymes Impairs Myogenesis

**Figure 2:**
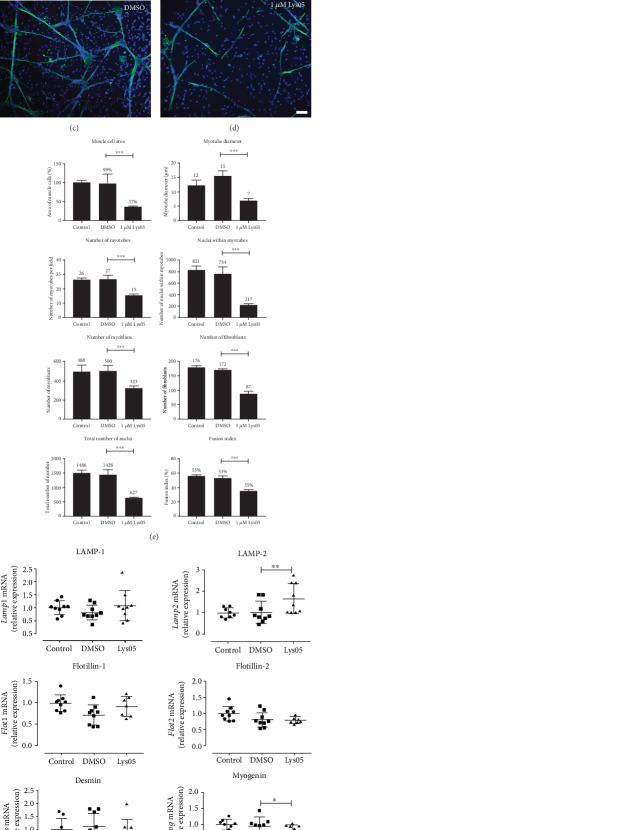
Inhibition of acidic compartment function impairs myogenesis and alters the expression of myogenic and lysosomal genes. Chick myogenic cells were grown for 24 hours, treated with Lys05 at 1 *μ*M, and double-labeled with an antibody against desmin and the nuclear dye DAPI (a–d). Several parameters of muscle proliferation and differentiation were analyzed (e). Image (a) shows the three different types of cells found in chick myogenic cultures: fibroblasts (F), myoblasts (Myb), and myotubes (Myot). Note that fibroblasts (F) are negative for desmin and have a larger nucleus (a), compared to myoblasts (Myb). Scale bar in (d) = 50 nm. qPCR was performed for flotillin-1, flotillin-2, LAMP-1, LAMP-2, desmin, and myogenin gene expression (f).

To further investigate the role of acidic compartments during chick myogenesis, we double-labeled Lys05-treated cells (1 *μ*M Lys05) for desmin and DAPI (Figures 2(b)–2(d)) and quantified parameters of muscle proliferation and differentiation in relation to fibroblasts, myoblasts, and myotubes.  Figure 2(a) shows the three different types of cells found in chick myogenic cultures: fibroblasts, myoblasts, and myotubes that were quantified. Note that fibroblasts are negative for desmin (Figure 2(a)). Lys05 induced a significant decrease in all the parameters of muscle proliferation and differentiation analyzed (Figure 2(e)): area occupied by muscle cells, diameter of myotubes, number of myotubes, number of nuclei within myotubes, number of myoblasts, number of fibroblasts, total number of nuclei, and fusion index. These experiments confirmed that inhibition of lysosomal function impairs myogenesis and suggests a role for acidic compartments and lysosomes in survival, proliferation, and differentiation of muscle cells.

### 3.4. Inhibition of Lysosomal Function Alters the Expression of Genes Related to Lysosomes and Muscle Differentiation

Next, we analyzed whether the effects of Lys05 on muscle cells involved alterations in gene expression. To answer that, we selected genes related to the lysosomal structure and function (LAMP-1 and LAMP-2 genes), early and late muscle differentiation (desmin and myogenin), and membrane recycling (flotillin-1 and flottilin-2). We analyzed the mRNA expression of these genes in untreated and Lys05-treated cells using qPCR. Lys05 treatment (for 24 hours) induced a significant decrease in the expression of myogenin mRNA levels and a significant increase in LAMP-2 expression (Figure 2(f)). No differences were found in the expression of flotillin-1, flotillin-2, LAMP-1, and desmin mRNA (Figure 2(f)). Curiously, LAMP-2 expression was the major alteration induced by Lys05 treatment. These results show that inhibition of lysosomal function by Lys05 induces changes in the expression of genes related to the lysosomal structure and function as well as in genes involved in muscle differentiation.

### 3.5. Knockdown of LAMP-2 Protein Inhibits Chick Myogenesis

**Figure 3:**
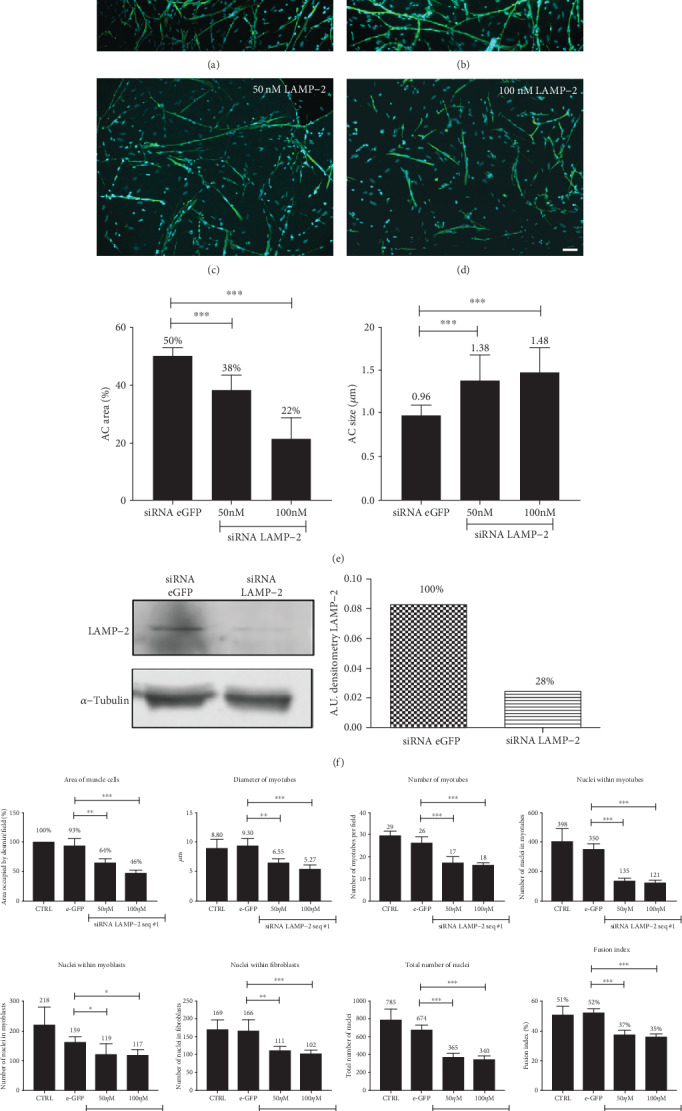
Knockdown of LAMP-2 protein inhibits chick myogenesis. Chick myogenic cells were transfected with siRNA against chick LAMP-2 or eGFP and double-labeled for desmin and DAPI (a–d). Scale bar in (d) = 50 *μ*m. Transfected cells were also submitted to Western blotting for LAMP-2 (f). Note that LAMP-2 knockdown induces a significant decrease in acidic compartment (AC) size, muscle proliferation, and differentiation (e, g).

Since we found that alterations in lysosomal function inhibits chick myogenesis and induces an increase in the expression of LAMP-2 (Figure 2), we decided to analyze the role of LAMP-2 during chick myogenesis. siRNA against *Gallus gallus* LAMP-2 was designed and transfected into chick myogenic cells. For control siRNA (nonspecific), we decided to target the enhanced green fluorescent protein (eGFP). Transfections with siRNA against LAMP-2 resulted in a reduction in the area of muscle fibers and produced a smaller amount (~70% decrease) of LAMP-2 protein, as well as reduction in the acidic compartment area and an increase in acidic compartment size (Figures 3(a)–3(e)). Remarkably, LAMP-2 knockdown induced a significant decrease in all the parameters of muscle proliferation and differentiation analyzed (Figure 3(g)): area occupied by muscle cells, diameter of myotubes, number of myotubes, number of nuclei within myotubes, number of myoblasts, number of fibroblasts, total number of nuclei, and fusion index. These results show that downregulation of LAMP-2 impairs chick myogenesis.

### 3.6. Activation of the Wnt/Beta-Catenin Pathway Rescues the Effects of Lysosomal Inhibition in Chick Muscle Cells

**Figure 4:**
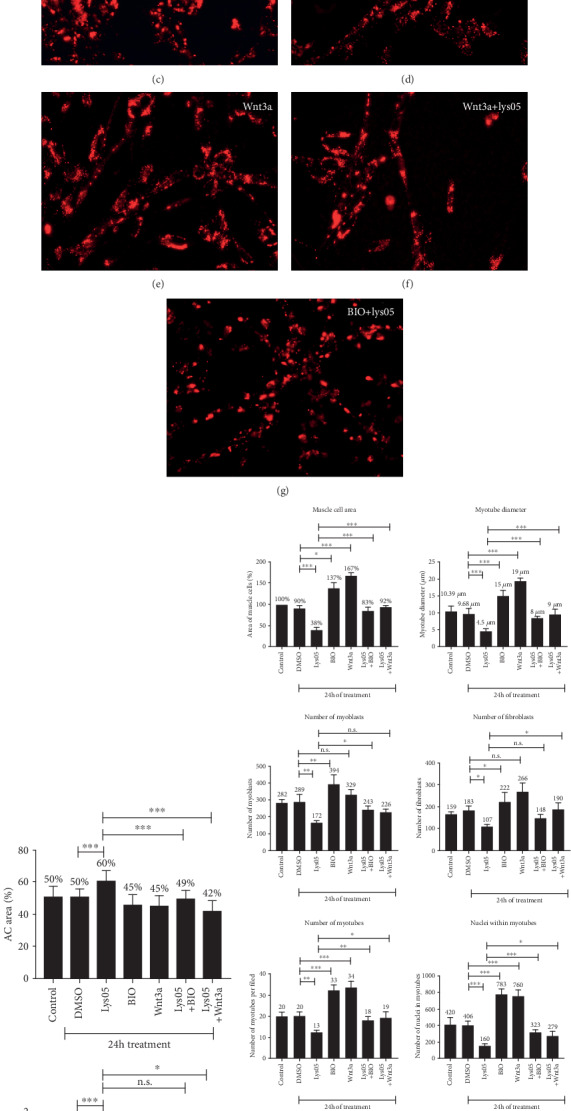
Activation of the Wnt/beta-catenin pathway rescues the effects of acidic compartment inhibition. Chick myogenic cells were treated with either DMSO, Lys05, BIO, or Wnt3a and labeled with acridine orange (a–g). Scale bar in (a) = 10 *μ*m. The area and diameter of acidic compartments (AC) were quantified (h). Cells were also labeled for desmin and DAPI, and several parameters of muscle proliferation and differentiation were quantified (i).

Our results showing a transitory increase in the area occupied by acidic compartments in 48 and 72 h cultures, in comparison with 24 and 96 h cultures (Figure 1), suggest that acidic compartment function is finely modulated during chick myogenesis. To gain an insight into acidic compartment regulation in chick muscle cells, we hypothesized that this process could be regulated by specific signaling pathways. Since the Wnt/beta-catenin signaling pathway has been shown to be involved in the regulation of myogenesis [6–8], we decided to test its possible involvement in acidic compartment function in myogenic cells. In the last years, our group has shown that the Wnt/beta-catenin pathway is activated when chick myogenic cells are treated with either a conditioned medium enriched in Wnt3a (obtained from L-cells) or BIO (a specific inhibitor of GSK3b), which are both activators of the Wnt/beta-catenin pathway [7]. These experiments were obtained by the transfection of chick myogenic cells with plasmids pGal (for b-galactosidase expression), FOPFlash (negative control luciferase reporter mutated in the TCF/Lef binding site), and TOPFlash (luciferase reporter containing the TCF/Lef binding site) and following the activation of the Wnt/beta-catenin signaling pathway by luciferase activity [7]. Here, we used BIO and Wnt3a to test the participation of the Wnt/beta-catenin pathway in lysosomal dynamics in chick muscle cells (Figure 4). Myogenic cells were treated with Lys05, BIO, Wnt3a, or Lys05 and labeled with acridine orange to visualize and quantify the area and diameter of acidic compartments (Figures 4(a)–4(h)). While Lys05 induced an increase in the acidic compartment area and diameter, BIO alone or Wnt3a alone did not significantly alter these parameters (Figure 4(h)). Interestingly, when BIO or Wnt3a were added together with Lys05, they prevented the increase in the acidic compartment area induced by Lys05 alone (Figure 4(h)). Curiously, cells treated with Lys05, Wnt3a, BIO, and Wnt3a together with Lys05 showed an accumulation of acidic compartments in the juxtanuclear region of myoblasts, fibroblasts, and myotubes (Figures 4(c)–4(g)). We also analyzed the relationship between the Wnt/beta-catenin pathway and acidic compartment function during muscle proliferation and differentiation (Figure 4(i)). Myogenic cells were treated with Lys05, BIO, Wnt3a, or Lys05 and labeled for desmin and DAPI. Our results showed that Lys05 induced a significant decrease, in comparison with DMSO-treated cells, in all the parameters of muscle proliferation and differentiation analyzed (Figure 4(i)), whereas cells treated with BIO alone or Wnt3a alone showed an increase in most of these parameters (Figure 4(i)). Interestingly, Lys05 and BIO together, as well as Lys05 and Wnt3a together, showed similar values of muscle proliferation and differentiation as the ones found in DMSO-treated cells (Figure 4(i)). These results suggest that the Wnt/beta-catenin pathway interferes with acidic compartment positioning and function in chick myogenic cells, as it inhibits and/or compensates the effects of Lys05.

**Figure 5:**
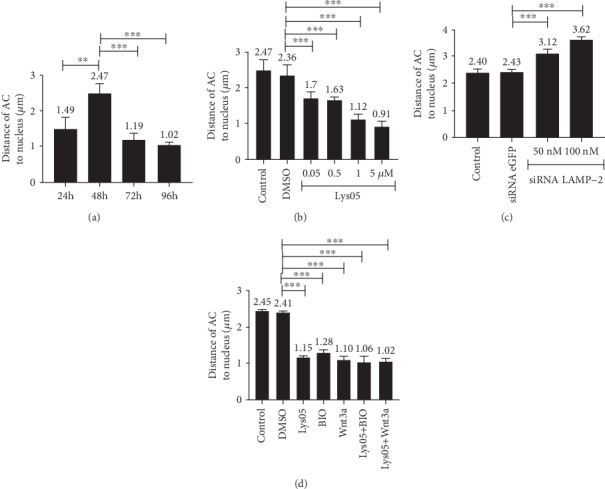
Acidic compartment positioning changes during the differentiation of chick myogenic cells and after disturbances in lysosomal function. Chick muscle cells were treated with DMSO, Lys05, BIO, and Wnt3a or transfected with siRNA to LAMP-2 or eGFP. Live cells were labeled with acridine orange, and the distance between acidic compartments (AC) and the nuclei of the cells was quantified (a–d).

To analyze whether the positioning of acidic compartments in the juxtanuclear region was somehow dependent on the Wnt/beta-catenin pathway, we calculated the distance between acidic compartments and nuclei in myogenic cells. Quantification of control untreated cells (with 24, 48, 72, and 96 hs) and treated cells (with DMSO, Lys05, BIO, Wnt3a, siRNA LAMP-2, and siRNA eGFP) is shown in Figure 5. Interestingly, 48 h cultures revealed to be the developmental stage in which acidic compartments were found more distant to nuclei, when compared to 24, 72, and 96 h (Figure 5). Treatment with Lys05 induced acidic compartments to be positioned closer to the nuclei of cells, and this effect was found to be concentration dependent (Figure 5). Cells transfected with siRNA against LAMP-2 exhibited acidic compartments more distant from the nuclei than cells transfected with siRNA against eGFP (Figure 5(c)). Cells treated with BIO or Wnt3a showed acidic compartments closer to the nuclei when compared to DMSO-treated cells (Figure 5(d)) and with similar values as compared to cells treated with 1 *μ*M of Lys05 (Figure 5(d)).

## 4. Discussion

Here, we analyzed the role of acidic compartments during chick muscle differentiation. We found an increase in the area occupied by acidic compartments during chick *in vitro* myogenesis. Chick myogenic cultures are a robust model of myogenesis in which it is possible to follow the initial steps of muscle fiber formation. 24 h cultures are composed of fibroblasts and at least two populations of mononucleated myoblasts (proliferating myoblasts and postmitotic myoblasts); 48 h cultures are composed of fibroblasts, bipolar postmitotic myoblasts, and young multinucleated myotubes; 72 and 96 h cultures are composed of fibroblasts, bipolar postmitotic myoblasts, and mature multinucleated myotubes (which spontaneously contract). Our data suggests that acidic compartments may have a role in the initial phases of embryonic chick myogenesis (from 24 to 96 h), such as in myoblast survival, myoblast proliferation, and myoblast fusion and in the formation of muscle fibers. These results are in accordance with previous data describing that LysoTracker fluorescence, GFP-LC3B puncta, and LC3B-II protein increase during the differentiation of the mouse myogenic cell line C2C12 [14].

In our work, we interfered with acidic compartment function by two ways: with the chloroquine Lys05 and with the downregulation of the major lysosomal protein LAMP-2 with siRNA. Both strategies lead to an inhibition of myogenesis. Lys05 and LAMP-2 siRNA induced a reduction in the number of myogenic cells and inhibited myoblast fusion, and the few myotubes formed were reduced in size, compared to untreated cells. Our results using LAMP-2 siRNA are in agreement with a recent report showing that LAMP-2 depletion impaired the differentiation of C2C12 myoblasts and reduced the diameter of myotubes [9]. These data together with ours suggest that acidic compartments are required for muscle differentiation.

The inhibitor of lysosomal function Lys05 induced a decrease in the number of myoblast cells and in the size of myotubes in chick myogenic cultures, which was accompanied by a decrease in the expression of myogenin mRNA levels. These results support a view of Lys05 as an inhibitor of chick myogenesis and reinforce an essential role of acidic compartments during the initial steps of chick muscle differentiation. Intriguingly, Lys05 induced an increase in the expression of LAMP-2. The increase in LAMP-2 expression can be correlated with the increase in acidic compartment diameter and area observed after Lys05 treatment. These results can be explained as Lys05 inhibiting acidic compartment function and cells responding by increasing their acidic compartments.

Interestingly, we found that Lys05 promotes the deacidification of acidic compartments in chick muscle cells while inducing an increase in the size and area of the acridine orange-labeled cellular compartments. Lys05 could be inducing the fusion of previously acidic cellular compartments (such as lysosomes) with other organelles involved with cellular recycling, such as late endosomes, multivesicular bodies, and intracellular vesicles coming from the plasma membrane and/or the Golgi/endoplasmic reticulum/mitochondria/nucleus membrane systems. Further studies are necessary to unravel the mechanisms involved in the deacidification and enlargement of acridine orange-labeled compartments in chick muscle cells after Lys05 exposure.

We found acidic compartments concentrated in the perinuclear region of cells during muscle differentiation. It has been described that in cultured rat kidney cells, lysosomes are typically distributed in the juxtanuclear region with a few additional lysosomes scattered throughout the cytoplasm [15]. These authors show that lysosomes accumulate in the region around the microtubule-organizing center (MTOC) and that this clustering of lysosomes depends on microtubules. The motility and positioning of lysosomes have been correlated with cell physiology and pathology [16]. The position of lysosomes within the cell determines their luminal pH: peripheral lysosomes are less acidic (and have impaired proteolytic activity) than juxtanuclear ones [17]. Therefore, the perinuclear localization of acidic compartments that we found in chick myogenic cells could be correlated with an increased acidic compartment activity in these cells.

Interestingly, we find that two activators of the Wnt/beta-catenin pathway, BIO and Wnt3a, were able to rescue the inhibitory effects of Lys05 in myogenesis, suggesting a role for the Wnt/beta-catenin pathway in the regulation of acidic compartment positioning and function in skeletal muscle cells. It has been shown that the canonical Wnt/beta-catenin signaling is a major regulator of endocytosis and that treatment of HeLa cells with Wnt3a induces a large increase in lysosomes [18]. Previous reports have shown that Wnt3a induces the endocytosis of several molecules related to the Wnt/beta-catenin pathway (including Axin, beta-catenin, Dvl-2, Frizzled, GSK3*β*, Lrp5/6, and Wnt) into late endosomes/multivesicular bodies/lysosomes [19, 20]. The internalization in endocytic vesicles of molecules involved in the Wnt/beta-catenin pathway is a molecular mechanism for controlling the on/off state of the signaling pathway. Interestingly, both BIO and Wnt3a induced an accumulation of acidic compartments in the perinuclear region of chick myogenic cells, whereas Lys05 induced a more dispersed distribution of acidic compartments within the cytoplasm of muscle cells. The accumulation of acidic compartments in the perinuclear region of chick myogenic cells that we observed after exposure of muscle cells to Wnt3a or BIO could be related to an increase in the internalization of Wnt-related molecules. We suggest that the activation of the Wnt pathway (by BIO and Wnt3a) induces the internalization of Wnt-signalosome complexes (including Axin, beta-catenin, Dvl-2, Frizzled, GSK3*β*, Lrp5/6, and Wnt) into acidic compartments, which are transported to the nuclear vicinity. In the juxtanuclear area, Wnt signaling molecules, such as GSK3*β*, will be degraded within active acidic compartments. In the absence of GSK3*β*, beta-catenin will be able to enter the nucleus and regulate the transcription of genes related to muscle differentiation. In contrast, the inhibition of lysosomal function (by Lys05) deacidifies acidic compartments, causing the impairment of lysosomal enzymes and leading to the accumulation of undergraded substrates in the acidic compartment lumen, which are found scattered in the whole cytoplasm of myogenic cells.

Importantly, in the present study, we did not analyze the role of autophagy during chick myogenesis. Our group intends to pursue a study to unravel the role of autophagy during the early developmental events of normal embryonic chick skeletal myogenesis, such as myoblast proliferation, migration, alignment, and fusion, as well as in myofibrilogenesis, sarcomere contraction, and muscle fiber growth.

The collection of results presented here highlights the role of acidic compartments during the initial steps of skeletal muscle differentiation and shows for the first time the participation of the Wnt/beta-catenin signaling pathway in acidic compartment size, positioning, and function during myogenesis.

## Data Availability

The authors confirm that the data supporting the findings of this study are available within the article and its supplementary material.

## Supplementary Materials

Supplementary Materials Supp. Fig. 1: sequences of Gallus gallus primers used in this study.
