# Supplementary material for: Acidic Compartment Size, Positioning, and Function during Myogenesis and Their Modulation by the Wnt/Beta-Catenin Pathway
Source: Biomed Res Int. 2020 Jun 20;2020:6404230. doi: 10.1155/2020/6404230 (PMC7322607; doi:10.1155/2020/6404230)
Supplement: Supplementary Materials — Supp. Fig. 1: sequences of Gallus gallus primers used in this study. [file 6404230.f1.zip › mat.6404230.v3.pdf]

Suppl. Fig. 1

DES

Des-F GAGCGTGACAACCTGCTAGA

Des-R CCACGTCAGCTCTGAAAGCA

Flotillin-1

Flot1-F CGGTGGAGGAGATCTATAAGGAC

Flot1-R CCCAGAGAGTGCAGGTAGTC

Flotillin-2

Flot2-F CACCCAGAGGAACCCTGACG

Flot2-R TCTCGATACCCATGCGACCC

GAPDH

GAPDH-F CAGAACATCATCCCAGCGT

GAPDH-R CAGGTCAGGTCAACAACAG

LAMP-1

Lamp1-F TCCTGCTCGGCTTTTTACAGG

Lamp1-R CACGAGCTGTGTGACTGTGA

LAMP-2

Lamp2-F AGAATGGTTCTGTCATTTTAAGTGC

Lamp2-R GCCAACTGCGACTGGAATAA

Myogenin

Miog-F AGCCTTCGAGGCTCTGAAAC

Miog-R CTGGGTGCAGCAGGTTGT
