# Supplementary material for: Acidic Compartment Size, Positioning, and Function during Myogenesis and Their Modulation by the Wnt/Beta-Catenin Pathway
Source: Biomed Res Int. 2020 Jun 20;2020:6404230. doi: 10.1155/2020/6404230 (PMC7322607; doi:10.1155/2020/6404230)
Supplement: Supplementary Materials — Supp. Fig. 1: sequences of Gallus gallus primers used in this study. [file 6404230.f1.zip › Supp Fig 1 Mermelstein_BMRI_3082499.doc]

**Suppl. Fig. 1**

**DES**

Des-F	GAGCGTGACAACCTGCTAGA

Des-R	CCACGTCAGCTCTGAAAGCA

**Flotillin-1**

Flot1-F	CGGTGGAGGAGATCTATAAGGAC

Flot1-R	CCCAGAGAGTGCAGGTAGTC

**Flotillin-2**

Flot2-F	CACCCAGAGGAACCCTGACG

Flot2-R	TCTCGATACCCATGCGACCC

GAPDH

#### GAPDH-F CAGAACATCATCCCAGCGT

#### GAPDH-R CAGGTCAGGTCAACAACAG

**LAMP-1**

Lamp1-F	TCCTGCTCGGCTTTTTACAGG

Lamp1-R	CACGAGCTGTGTGACTGTGA

**LAMP-2**

Lamp2-F	AGAATGGTTCTGTCATTTTAAGTGC

Lamp2-R	GCCAACTGCGACTGGAATAA

**Myogenin**

Miog-F	AGCCTTCGAGGCTCTGAAAC

Miog-R	CTGGGTGCAGCAGGTTGT
